# A new species of *Oxyptilus* Zeller from the southwestern United States (Lepidoptera, Pterophoridae)

**DOI:** 10.3897/zookeys.698.14999

**Published:** 2017-09-18

**Authors:** Deborah L. Matthews

**Affiliations:** 1 McGuire Center for Lepidoptera and Biodiversity, Florida Museum of Natural History, University of Florida, P.O. Box 112710, Gainesville, Florida 32611-2710, USA

**Keywords:** Davis Mountains, *Crombrugghia*, *Hieracium*, Madrean Archipelago, Nearctic Region, new species, Paliza Canyon, sky islands

## Abstract

*Oxyptilus eleanerae* **sp. n.**, is described from New Mexico, Arizona, and Southwest Texas, and compared with the only other *Oxyptilus* occurring in the Nearctic Region, *O. delawaricus* Zeller. A redescription is provided for *O. delawaricus*. Adults and male and female genitalia are illustrated for both species. Key morphological characters, distributions, and relationships within the genus are discussed.

## Introduction

The genus *Oxyptilus* Zeller (type species *Pterophorus pilosellae* Zeller) formerly included 28 species worldwide (Gielis 2003). A recent review and phylogenetic analysis of the tribe Oxyptilini (Alipanah et al. 2011) recovered only five species [*O. pilosellae* (Zeller), *O. parvidactyla* (Haworth), *O. chrysodactyla* (Denis and Schiffermüller), *O. delawaricus* Zeller, and *O. ericetorum* (Stainton)] as a monophyletic group referred to as “true Oxyptilus,” with the genus *Crombrugghia* Tutt recognized as the sister group to *Oxyptilus* within the tribe. The five “true *Oxyptilus*”, including the Nearctic species, *O. delawaricus*, are associated with the composite genus *Hieracium* as larval hosts (Gielis 2003, Matthews and Lott 2005). Known hosts of *Crombrugghia* likewise include *Hieracium* as well as related composites.

Of the remaining 28 species listed by Gielis (2003), some have been assigned to other genera (Alipanah et al. (2011), while others were not available for study. Alipanah et al. (2010) concurrently described two new genera (*Apoxyptilus* Alipanah et al. and *Pseudoxyptilus* Alipanah et al.) for outlying taxa studied which were previously included in *Oxyptilus* and provided a decisive key to the 19 genera now included in the tribe Oxyptilini.

The Nearctic fauna includes eight genera of Oxyptilini. Of these, *Dejongia* Gielis, *Trichoptilus* Walsingham, *Megalorhipida* Amsel, and *Buckleria* Tutt can be easily separated from the others by the narrow, apically pointed lobes of the forewing which lack a distinct termen. Following key characters described and illustrated in Alipanah et al. (2010), *Geina* Tutt and *Capperia* Tutt can be separated from *Sphenarches* Meyrick and *Oxyptilus* by the more posterior (as opposed to central) position of a concavity in the hourglass-shaped tergite of the second abdominal segment in males (Figs 27, 29). Finally, *Oxyptilus* is distinguished from *Sphenarches* in having a scale brush on the second (middle) segment of the labial palpus which extends ventrally along the first (apical) segment.

Several years ago, while identifying and curating Pterophoridae at the McGuire Center, I found three very worn specimens from the Davis Mountains of Texas that I recognized as something different and confirmed by dissection that they were not *O. delawaricus* as previously determined. Although recognizing the species as new, I did not proceed with further investigation until James Adams presented me with a fresh female for identification from samples he and his mom collected on a memorable trip to the Davis Mountains in 2005. A recent trip to the National Museum of Natural History, Washington, D.C. resulted in the location of additional material from Arizona and New Mexico within pro-tem holdings. In addition, the 2016 donation of the Texas Lepidoptera Survey collection to the McGuire Center by Ed Knudson and the late Charles Bordelon yielded six more specimens from the Davis Mountains. This new species was confirmed as a “true Oxyptilus” through key characters in Alipanah et al. (2010) and is described and distinguished from *O. delawaricus*. CO1 barcodes from two USNM specimens further support the distinct identity and placement within the genus.

## Material and Methods

Specimens examined for this study, including comparative material of *O. delawaricus*, are from the following institutions and private collections: Academy of Natural Sciences of Philadelphia (**ANSP**); Deborah Matthews Collection, Gainesville, Florida (**DMC**); Canadian National Collection, Ottawa (**CNC**); Great Lakes Forestry Centre, Sault Ste. Marie, Ontario (**GLFC**); Laurence L. Crabtree Collection, Adin, California (**LLCC**); Museum of Comparative Zoology, Harvard University (**MCZ**); McGuire Center for Lepidoptera and Biodiversity, Florida Museum of Natural History, University of Florida (**MGCL**); Florida State Collection of Arthropods, Lepidoptera housed at McGuire Center for Lepidoptera and Biodiversity, Gainesville, Florida (**FSCA**); Mississippi Entomological Museum, Mississippi State, Starkville, Mississippi (**MEM**); Essig Museum of Entomology, University of California, Berkeley, California (**UCB**); National Museum of Natural History, Washington, D.C. (**USNM**); Department of Entomology, Washington State University, Pullman, Washington (**WSU**). Collections or institutions are indicated within material examined by the database number prefix or the collection coden given in parentheses if the specimen has not yet been assigned a unique identifier.

Genitalia were prepared following standard dissection techniques of tissue maceration in heated 10% KOH and light staining with Chlorazol Black E and Eosin Y followed by slide mounting in Euparal. Adult images were taken with a Canon 70D camera and Canon 100mm IS macro lens against a standard gray background in a white reflective light funnel illuminated with OttLite bulbs. Genitalia slides were photographed at manually selected multiple focal planes using a Canon Rebel T3i camera mounted on a Zeiss Axiophoto transmitted light microscope in conjunction with Canon EOS Utility software. Ventral adult images and genitalia images were stacked as needed with Zerene Stacker, version 1.04 using the DMap algorithm and images were assembled on the plates with Adobe Photoshop CS5.1. Images of heads and palpi were taken with an Auto-montage Pro 5.01 system (Synoptics Ltd.) using a JVC digital camera (model KY-F65U) and Leica Z16APO lens.

Forewing measurements in descriptions are from the wing base to apex and include apical fringe scales. Cleft origin proportions exclude fringe scales. Wing venation follows Miller (1970). Colors in descriptions follow Ridgway (1912). CO1 barcodes and the taxon identity tree discussed were retrieved via the public portal of Bold Systems (Ratnasingham and Hebert 2007).

### Oxyptilus eleanerae

Taxon classificationAnimaliaLepidopteraPterophoridae

Matthews sp. n.

http://zoobank.org/5F95486F-EDF6-4F7F-8E60-674BC758D583

Figs 1–9 , 13 , 14 , 19 , 20 , 22–24 , 29 , 30

#### Type material.

**HOLOTYPE.** ♀ – with the following labels: ΄U.S.A. NM: Sandoval Co. │ 1.1 mi NE jct. 10 & 266 │ on 266, 7319 ft. │ 35.70860° N 106.61876° W │27 July 2013 MA Solis΄ [white printed]; ΄HOLOTYPE ♀ │ *Oxyptilus* │ *eleanerae* │ D. Matthews΄[red printed]; ΄USNMENT │ 01338013΄ [white thermal printed with data matrix code]. **PARATYPES**. 13 ♂, 20 ♀ as follows: 4 ♂ – USA: ARIZONA: Apache Co.: White Mts., 7200 ft, 1 – 15 Aug 1925, O.C. Poling, USNMENT01338024, 01338029 [slide DM 1822], 01338031, 01338033; 2 ♀ – same data, USNMENT01338022, 01338032 [slide DM 1835]; 2 ♂ – same data except, 1–15 Sep 1925, USNMENT01338015 [slide DM 1817], USNMENT01338026; 1 ♀ – same data, USNMENT01338027; 1 ♂ – White Mts., near McNary P.O., 15 – 30 Aug 1925, O.C. Poling, USNMENT01338028; 1 ♀ – same data, USNMENT01338030; 1 ♀ – White Mts., near Rice, 7000 ft, 15 – 30 Jul 1925, O.C. Poling, USNMENT01338025 [slide DM 1831]; 1 ♀ – Coconino Co.: Chiricahua Mts., Herb Martyr forest camp, 5840 ft, 7 Aug 1966, Robert G. Beard, at UV light, Barcode of Life DNA voucher specimen, SmplID CCDB-20275-B04, BOLD Proc. ID LNAUS2296-13, USNMENT00869147; 1 ♂ – Clover Springs, 25 Aug 1978 R. Wielgus, CUVBL, Barcode of Life DNA voucher specimen, SmplID CCDB-20275-B05, BOLD Proc. ID LNAUS2297-13, USNMENT00869148; 1 ♂ – Fort Valley, 7 ½ mi. NW Flagstaff, 7350 ft, 16 Aug 1961, Ronald W. Hodges, USNMENT01338020; 1 ♀ – Hochderffer Hill, 12 ½ mi. NNW Flagstaff, 8500 ft, 4 Aug 1961, Ronald W. Hodges, USNMENT01338017; 1 ♀ – Kehl Spring Forest Camp, 7450 ft, 30 Jul 2014, R.S. Wielgus, 15 watt UV light trap, USNM [no number]; 1 ♂ – West Fork, 16 mi. SW Flagstaff, 6500 ft, 13 Jul 1961, Ronald W. Hodges, USNMENT01338016 [slide USNM PYR. 93 / RWH USNM 63,602]; 1 ♀ – same data except 13 Aug 1961, USNMENT01338023; 2 ♀ – same data except 20 Aug 1961, USNMENT01338019, 01338021; 1 ♀ – County unspecified [locality near Santa Cruz/Pima border]: Madera Canyon, Santa Rita Mtns., 4880 ft, 29 Jul 1959, Ronald W. Hodges, USNMENT01338018; 1 ♂ – NEW MEXICO: Sandoval Co.: Valles Caldera National Preserve, 0.5 mi. past jct. VC033 & VC06 on VC06, 9610 ft, 35.8994°N, 106.5670°W, 24 Aug 2010, M. Pogue & M. Metz, collected in MV light trap 3, USNMENT01338014 [slide DM 1829]; 1 ♂ – TEXAS: Jeff Davis Co.: Davis Mtns. Resort, 5800 ft, 29 Sep 1994 D.G. Marqua, Acc. No. 2009-30, MGCL 100049 [slide DM 1823]; 1 ♀ – same data except 21 Jul 2004, MGCL 168736 [slide DM 1812]; 1 ♀ – Davis Mts. Pres., Madera Canyon 5800’ 12,13 Sep 2001 B/K [Bordelon/Knudson] (MGCL); 1 ♂ – Davis Mts. State Park 27 Jun – 1 Jul 1987 J.B. Heppner, MGCL 168735 (FSCA) [slide DM 1613]; 3 ♀ – same location, 22,23 Aug 1995, E. Knudson (MGCL); 1 ♀ – same location 3 Oct 1999 ECK [Knudson] (MGCL); 1 ♀ – Ft. Davis, 19 Aug 1984 E. Knudson (MGCL); 1 ♀ – 18.5 road mi. NW of Fort Davis, along St. Hwy. 118, 10–11 Aug 2005 James & Eleaner Adams, light trap, MGCL 168734.

#### Deposition of types.

The holotype and 23 paratypes are property of the National Museum of Natural History, Washington, DC. (USNM). In addition, ten paratypes are deposited at the McGuire Center for Lepidoptera and Biodiversity (MGCL), one of which [MGCL 168735] is the property of the Florida State Collection of Arthropods (FSCA) housed within the McGuire Center.

#### Additional material.

1 ♂ – USA: Arizona: Pima Co. Santa Rita Mts., Madera Canyon 17 Aug 1949 C.W. Kirkwood, CPK collection, slide DM 634 (MCZ). This specimen is identified as *O. eleanerae* based on images of the genitalia slide. The specimen was previously examined by the author and returned to MCZ identified as *O. delawaricus*. This specimen is excluded from the type series as it was not on hand at the time of preparation of the present description.

#### Diagnosis.

This species is distinguished from the only other nearctic *Oxyptilus*, *O. delawaricus*, by the drab or grayish ground color as opposed to ochraceous-tawny in *O. delawaricus*. It is further distinguished by having white as opposed to ochraceous-tawny or clay colored apices on both ventral forewing lobes. The anterior dorsal forewing lobe has two transverse white bands, with the more basal band distinctly wider as opposed to similar widths in *O. delawaricus*. The hindwing second lobe has a distinct patch of white linear fringe scales two-thirds from the wing base along the anal margin which only appears as a trace in some *O. delawaricus*. In *O. eleanerae*, the ventral surface of the abdomen has a strong mesal band, about twice the width of that in *O. delawaricus*. Key differences in the male genitalia include distinctly shorter tegumen lobes in males of *O. eleanerae* and the bilobed process of sternite VIII with triangular as opposed to finger-shaped lobes. Females of *O. eleanerae* have proportionally larger and robust signa and simple cup-shaped antrum without a dorsal bilobed marginal lip.

#### Description

**Figures 1–12.**
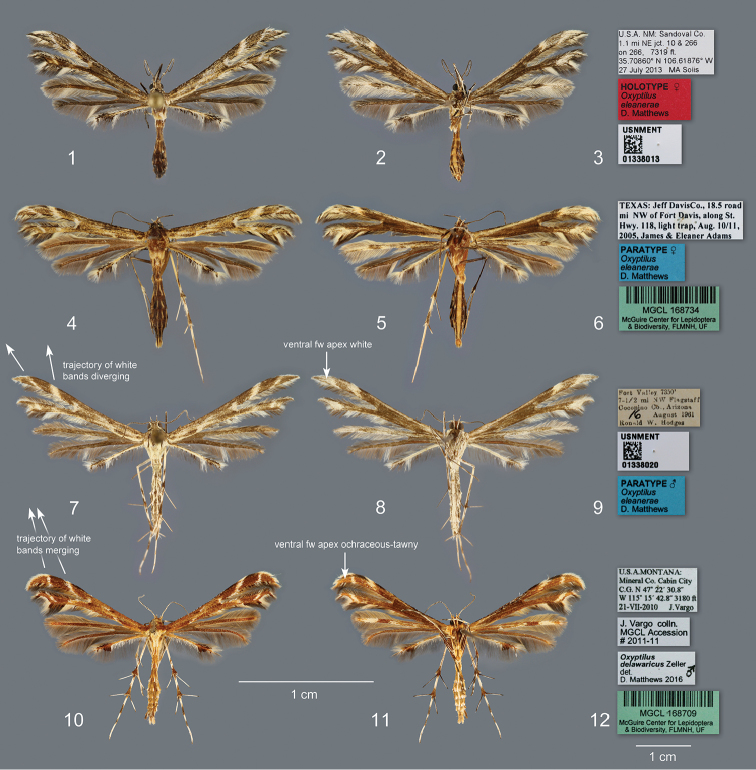
*Oxyptilus* adults and corresponding labels: **1** dorsal view of *O. eleanerae*, holotype, female, New Mexico, Sandoval County **2** ventral view of holotype **3** holotype specimen labels **4** dorsal view of female paratype, Texas, Jeff Davis County **5** ventral view of female paratype **6** female paratype labels **7** dorsal view of male paratype, Arizona, Coconino County **8** ventral view of female paratype **9** male paratype labels **10** dorsal view of male *O. delawaricus*, Montana, Mineral County **11** ventral view, same individual **12** specimen labels.

**Figures 13–16.**
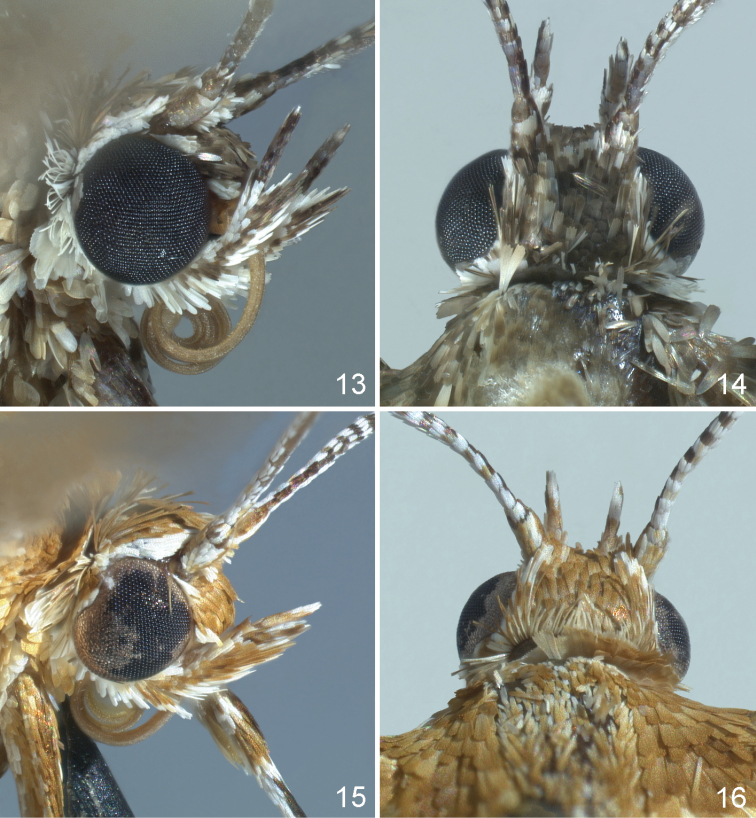
Close up of *Oxyptilus* heads: **13** lateral view of *O. eleanerae*, holotype **14** dorsal view of holotype **15** lateral view of *O. delawaricus* (same specimen as figure 10) **16** dorsal view of same specimen.

(male, female). Based on the holotype (female) and 33 paratypes (13 males, 20 females). HEAD (Figs 13–14) with labial palpi slender, length 1.5× eye diameter. Second segment with ventral scale tuft reaching one-third to half of third (distal) segment. Palpi white and drab or chestnut-brown with middle segment white with drab lateral stripe; distal segment usually white dorsally, drab ventrally. Frons and vertex drab with scattered white scales, front with narrow white band just anterad of antennae and another narrow white band along anterior margin. Eye bordered by narrow ring of white appressed scales. Occipital fringe scales bifid or trifid, mixed drab and white, mostly white dorsally, drab and longer laterally, white ventrally close to eye. Antenna with scape and pedicel with three white and three chestnut-brown or drab alternating stripes; flagellum drab or chestnut-brown dorsally, dotted with white scales; drab or chestnut-brown, minutely ciliate ventrally. THORAX with anterior half of tegulae and mesoscutum covered with mixture of drab and cream, drab-tipped scales. Posterior half of tegulae, mesoscutum, and mesoscutellum white to cream colored; metascutum with weak medial and subdorsal patch of buff scales flanked by white stripes. Foreleg (Fig. 2) chestnut-brown and white striped except coxa proximally white, distal surface solid drab or chestnut-brown; tibia with variably white and chestnut-brown scaled tuft at epiphysis; tarsomeres variable, mostly drab or chestnut-brown dorsally, pale drab ventrally, distal ends of each segment somewhat darker. Midleg coxa striped with buff and cream colored scales; femur and tibia chestnut-brown and white striped, venter mostly white, tibial spurs subequal, dorsally white, ventrally chestnut-brown, scale tuft laterally white, proximally chestnut-brown, tarsomeres as on foreleg. Hindleg coxa as on midleg, femur with double chestnut-brown stripe laterally, tibia mostly white with a narrow oblique chestnut-brown stripe and solid band preceding spurs. Spurs equal, white dorsally, chestnut-brown ventrally, length just less than that of first tarsomere. Tarsomeres strongly banded with distal part of each chestnut-brown. FOREWING (Figs 1–2, 4–5, 7–8) length, males xˉ = 10.35 mm ± 0.59 (n = 13), females, xˉ = 10.30 mm ± 0.76 (n = 15), holotype 10.50 mm. Cleft origin 0.49–0.53× wing length from base, lobe apices acute, second lobe with concave termen. Dorsal ground color appearing drab to grayish-olive at a distance, composed of mixed drab and pale orange-yellow to light orange-yellow scales. Costal margin drab mixed with chestnut-brown. Discal cell with small central chestnut-brown spot. Area between veins 1A and 2A with diffuse elongate patch of chestnut-brown scales near wing base surrounded by white scales. Cleft base marked with white crescent-shaped patch subtended basally at M_2_ by small chestnut-brown spot. First lobe transected in thirds by two oblique white bands, basal band distinctly wider and projecting at a more acute angle from the long axis of the wing than distal band. White scaling of bands extending to costa, interrupting darker scales of costal margin. Second lobe similarly transected by white bands except basal band diffuse. Cleft margins of both lobes bordered by chestnut-brown to fuscous-black scales. Fringes mixed white, pale orange-yellow, and drab with a few spatulate scales of cleft margin overlapping linear fringe scales. Forewing anal margin with terminal and subterminal groups of drab linear fringe scales, the subterminal patch (between terminus of Cu_1_ and Cu_2_) larger and flanked by white patches of linear scales. Subterminal patch with scattered overlapping white and pale orange-yellow scales. Fringes at basal third of second lobe with small patch of 2–4 spatulate fuscous-black scales. Fringes of anal margin basad of cleft mostly drab, with distinct patch of white overlapping elongate spatulate white scales just basad of cleft. Ventral forewing drab except for white or cream colored border along costa, diffuse oblique white band across basal third of first lobe, and white scaling covering distal third of both lobes. HINDWING (Figs 1–2, 4–5, 7–8) first and second lobes dorsally uniform drab or chocolate brown with drab fringe except for patch of white linear fringe scales two-thirds from wing base along second lobe anal margin. Third lobe white or cream colored with row of drab scales along anterior margin and few scattered drab or fuscous-black scales along anal margin. Distal third of lobe with distinct patch of spatulate fuscous-black scales extending into fringes along anterior margin with scattered white scales. Third lobe anal margin with small patch of 4 or 5 drab or fuscous-black spatulate scales in fringes at apex and second larger patch of 10–20 fuscous-black spatulate and elongate scales at 0.23× from apex. A similar sized patch of white scales present just basad of the latter patch and including some white linear fringe scales. White linear fringe also present at lobe apex. Remaining linear fringe scales uniformly drab. Ventral hindwing with first lobe white except for drab or mixed drab and pale orange-yellow band covering central 0.2× of part of lobe beyond cleft. Area near and basad of cleft mixed white, drab, and pale orange-yellow. Fringes of first lobe mostly white, mixed with some drab linear scales distally. Second lobe drab with some scattered white scales near apex; fringes drab except for white patch along anal margin as on dorsum. Venous scales antique brown. Third lobe venter entirely white or white with drab margins on distal part near scale tooth. Third lobe fringes as on dorsum. ABDOMEN dorsum mottled drab and white, with white dorsal line diverging laterad toward posterior margin of segments A2–A6. Two broken, narrow white lines laterally on A2–A7. Abdomen venter with strong mesal white band, flanked posteriorly by white domed spots on A3–A6 and contiguous white band on A7–A8.

**Male genitalia** (Figs 19, 20) (n=6). Uncus bilobed, weakly sclerotized, mesally articulated with tegumen, longer than and extending distad of tegumen for most of length. Tegumen bilobed, lobes equal or shorter than valvular lobes, basally widened, distally rounded. Valvae symmetrical, with terminal membranous valvular lobe accounting for 0.25 – 0.40× entire length of valve. Valvular lobe bearing deciduous scale tuft and short setae, basal connection only slightly constricted. Sacculus well developed, distinctly wider in basal half. Juxta a short curved sclerotized fulcrum lacking anellus arms and fused with membranous sheath of phallobase. Phallus very large, length about 1.5× that of valvae, width at base equal or slightly greater than that of valvae, with slight bend at middle near connection point of juxta, dividing phallobase and aedeagus. Vesica tubular, without cornuti, preceded at tip of phallus by dense concentric arrangement of spiculae. Inception of bulbus ejaculatorius at about 0.5× length of phallobase. Sternite VIII produced as bilobed structure medially supported by the saccus, overall length similar to that of tegumen lobes, notch between lobes reaching about 0.3× distance to base. Lobes widened basally, appearing triangular.

**Female genitalia** (Figs 22–24) (n=3). Apophyses posteriores about 3.6× length of papillae anales, moderately sclerotized, anterior ends simple. Apophyses anteriores absent or represented by small laterally curved sclerotized area at anterolateral margin of tergite VIII. Sternite VII overriding VIII and forming recessed elongate irregularly shaped pocket in which ostium is centrally placed. Ostium a circular moderately sclerotized rim on simple cup-shaped antrum extending anterad of sternite pocket. Antrum length similar to ostial diameter. Ductus bursae and corpus bursae similar in length. Width of ductus bursae about 0.5× ostium diameter. Width of ductus seminalis similar or just less than ductus bursae, inception point with corpus bursae directly adjacent to that of ductus bursae. Corpus bursae ovoid, with paired oblong signa. Signa with central ridge and equal or just exceeding ostium diameter in length.

#### Etymology.

This species is named in honor and memory of Eleaner Ruth Adams who together with her son James, collected one of the paratypes in the Davis Mountains of Texas. Eleaner is fondly remembered for her sense of adventure and passion for natural history which she passed on to her sons and grandchildren. The epithet is a noun in the genitive case reflecting the meaning of the common name Eleaner’s *Oxyptilus*.

#### Larval hostplants and habits.

Unknown. Other species in the genus feed on *Hieracium*. Seven native species of *Hieracium* are known to occur within parts of the range of *O. eleanerae*. Of particular interest as a potential host is *Hieracium carneum* Green (Huachuca hawkweed) which has a similar range to *O. eleanerae*, restricted to Arizona, New Mexico, and Texas (USDA 2017) and also extends into Chihuahua, Mexico.

#### Distribution and phenology.

The holotype was collected in Sandoval County New Mexico, within the Santa Fe National Forest southeast of Jemez Springs and just east of Paliza Canyon. The area is dominated by ponderosa pine with some Douglas fir, oak brush, and nearby pinyon-juniper woodland. The habitat where the holotype was collected (based on an image provided by M.A. Solis) was a canopy gap adjacent to Highway 266. This opening was surrounded by *Pinus ponderosa*, a few *Populus tremuloides*, and shrub oaks. Ground cover included *Ericameria* (rabbit brush) in bloom and sparse grasses. Low groundcover also included some rosette leaf clusters which could be *Hieracium* as well as *Antennaria* but these cannot be identified with certainty from the photo. The altitude of the type locality is 2231 meters (7319 feet).

The known distribution (Fig. 31) covers parts of three states: New Mexico, Arizona, and a small area of western Texas restricted to the Davis Mountains. Locality elevations range from 1585 to 2929 meters (5,200 to 9,610 feet). These “sky island” habitats, with pine-oak woodlands and ponderosa pine, are isolated by surrounding desert. Populations of *O. eleanerae* are likely to be present further south in other Madrean Sky Islands of northwestern Mexico. Collection dates range from 27 June to 3 October with no detectable flight patterns based on material available. Interestingly, significantly more females have been collected than males and in most cases, samples are indicated as coming from light traps. No explanation for this imbalance is apparent.

**Figures 17–20.**
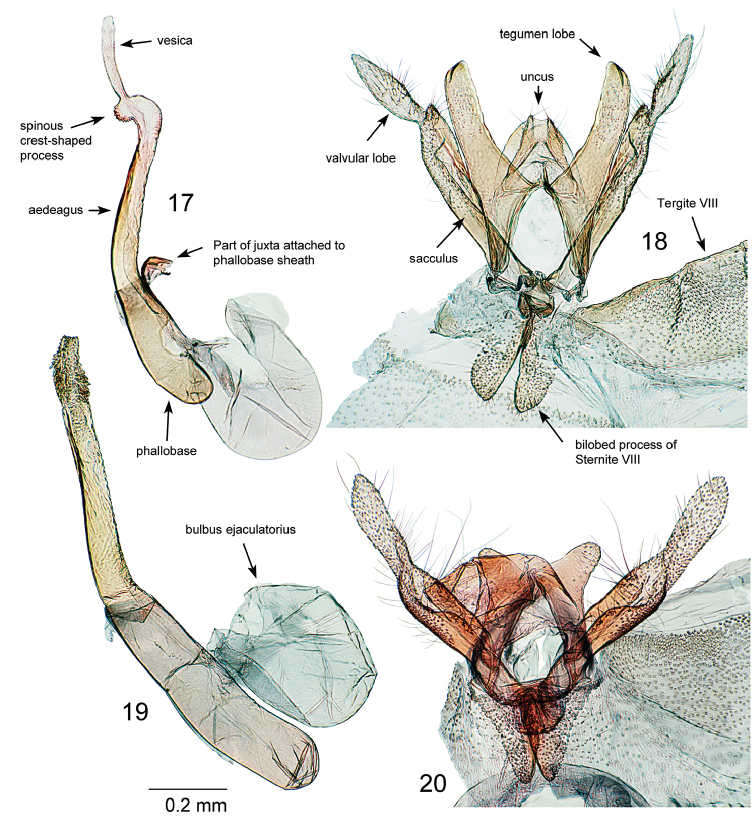
*Oxyptilus* male genitalia: **17** *O. delawaricus* phallus, slide DM 1837, California, El Dorado County, S. Lake Tahoe, Cold Creek, 10 Aug 1991, D.L. Bauer, MGCL Acc. # 2010-29 **18** same individual and slide, genitalia in situ at sternite VIII, with sternite VIII bilobed structure folded open toward anterior, and phallus removed **19** *O. eleanerae* male paratype, phallus, slide 1829, New Mexico: Sandoval County (see Type material section) **20** same individual, genitalia in situ at sternite VIII, with sternite VIII bilobed structure folded open toward anterior, and phallus removed.

### Oxyptilus delawaricus

Taxon classificationAnimaliaLepidopteraPterophoridae

Zeller, 1873

Figs 10–12 , 15–16 , 17–18 , 21 , 25–26 , 27–28

Oxyptilus delawaricus Zeller, Verhandlungen der Kaiserlich-Königlichen Zoologisch-Botanischen Gesellschaft in Wien Vol. 23, p. 320, 1873.
Oxyptilus bernardinus f. finitimus Grinnell, Canadian Entomologist, Vol. 19, p. 315, 1908.

#### Material examined.

More than 284 specimens were examined from various institutions and label data captured. In the interest of brevity, locality data is given in captions for figured specimens and summarized for all other *O. delawaricus* specimens examined in the distribution map (Fig. 31). In some cases, where only a state was given on labels and no other state records available, a dot was placed in the center of the state. An exception is Kansas where the dot was centrally placed in Eastern Kansas, reflecting the most likely distribution based on the known distribution of *Hieracium* within the state (USDA 2017).

#### Diagnosis.

As in *O. eleanerae*, the second segment of the labial palpus bears a scale tuft that extends partway alongside the distal segment distinguishing it from related genera. The species is recognized externally by the ochraceous-tawny ground color; the metascutum with three ochraceous-tawny or clay color stripes, as opposed to buff in *O. eleanerae* (mid-dorsal stripe may be obscure), and flanked by narrow white stripes with the pattern continued on the first abdominal segment; and patterns of the forewing first lobe. Dorsally, the first lobe is traversed by equally narrow white stripes, as opposed to a wider proximal band as in *O. eleanerae*. The trajectory of the distal white stripe is also at a smaller angle than in *O. eleanerae*. Ventrally, the forewing apex is ochraceous-tawny or clay colored as opposed to white in *O. eleanerae*. The midventral white band of the abdomen is narrow in *O. delawaricus*, about half the width of that in *O. eleanerae*. Genitalia characters also distinguish the two species including the longer tegumen lobes and constricted base of the valvular lobe in *O. delawaricus* males, and the bilobed ostial margin and smaller signa in females.

#### Redescription

(male, female). HEAD (Figs 15, 16) with labial palpi slender, length about 1.5× eye diameter. Second segment with ventral scale tuft reaching 0.3× to .0.75× of distal segment. Palpi white and clay colored, with clay colored lateral stripe. Basal segment mostly white, distal segment dorsally with white tip. Frons and vertex ochraceous-tawny or clay colored with transverse single row of white scales anterad of antennae and cluster of white scales laterally at anterior margin or across entire frontal margin. Eye bordered by white scales as in *O. eleanerae* except sometimes with clay colored scales mixed in. Occipital fringe scales mostly bifid, ochraceous-tawny with some white scales mid-dorsally. Antenna with scape and pedicel with three alternating white and brown stripes, anterior stripes chestnut-brown; posterior stripe ochraceous-tawny; flagellum dotted with alternating chestnut-brown and white scales dorsally, drab and minutely ciliate ventrally. THORAX with anterior part of tegulae and mesoscutum russet or ochraceous-tawny, grading posteriorly to clay, cream, and white; mesoscutellum mostly cream colored and white; metascutum with three ochraceous-tawny or clay colored stripes flanked by white stripes. Foreleg (Fig. 11), chestnut-brown and white striped, except coxa proximally white, distally ochraceous-tawny; femur evenly striped, tibia chestnut-brown dorsally, white ventrally; first tarsomeres with dorsal dusky-brown stripe, ventrally white, second and third tarsomere white and dusky-brown banded, fourth and fifth tarsomere solid dusky-brown. Mid and hindleg coxa ochraceous-tawny and white or cream striped; femur white and chestnut-brown striped; tibia white with chestnut-brown bands terminating in scale tufts at spurs. Spurs nearly equal, white dorsally with dusky-brown apex, dusky-brown to black ventrally. Tarsomeres white and dusky brown banded, somewhat variable, with distal tarsomere usually dusky-brown. FOREWING (Figs 10, 11) length, males xˉ = 9.58 mm ± 0.56 (n = 25), females, xˉ = 8.71 mm ± 0.80 (n = 6). Cleft origin 0.48–0.53× wing length from base, lobe apices acute, second lobe with concave termen. Dorsal ground color ochraceous-tawny. Costal margin dotted with white and chestnut-brown. Discal cell with small central chestnut-brown spot with distal trail of white scales. Area between veins 1A and 2A with diffuse elongate patch of russet and chestnut-brown scales. Cleft base with white spot with smaller chestnut-brown spot posterad. First lobe transected in thirds by two oblique narrow white bands of similar width; basal band projecting at more obtuse angle from long axis of wing than distal band. White scaling of bands extending to costa, with some ochraceous-tawny scales mixed in at margin. Forewing apex ochraceous-tawny, between white bands russet, area just basad of basal white band, russet, gradually grading basally to ochraceous-tawny. In some cases, forewing ground color appearing entirely russet with area between white bands not distinctly darker than apex. Second lobe similarly patterned to first except basal white band diffuse and area just basad ochraceous-tawny. Cleft margins of both lobes with scattered spatulate chestnut-brown scales, and elongate clay colored scales overlapping drab linear fringe scales; white spatulate scales likewise overlapping fringe at white bands. Forewing anal margin with terminal and subterminal groups of drab linear fringe scales as in *O. eleanerae*, the subterminal patch larger, flanked by white patches of linear scales, and with dense row of shorter overlapping russet and ochraceous-tawny scales. Fringes at basal third of second lobe with small patch of chestnut-brown and white. Fringes of anal margin basad of cleft mixed drab and white with patch of overlapping elongate white scales basad of cleft. Ventral forewing russet basad of cleft, with mixed ochraceous-tawny, clay, and white scales closer to wing base and trailing along narrow chestnut-brown costal margin. Russet scales grading into lobes to ochraceous-tawny, including lobe apices. White oblique bands as on dorsum except wider and with basal band of second lobe absent. Costal margin of first lobe with short conspicuous dusky-brown or chestnut-brown fringe, contrasting white fringe at basal band and apical third of lobe. Fringes of cleft margin mostly drab. Anal margin fringe alternating white and drab as on dorsum but with spatulate scales mostly hidden by linear fringe scales. HINDWING (Figs 10, 11) first and second lobes dorsally russet, basally grading to drab. Fringes drab, sometimes with trace of white two-thirds from wing base along second lobe anal margin. Third lobe ochraceous-tawny on basal two-thirds, russet on distal third adjacent to scale tooth, occasionally uniformly russet. Scale tooth at distal third of lobe with short spatulate russet and fuscous-black scales extending into fringes along anterior margin; anal margin with russet and fuscous-black in distal third forming triangular patch with basal scales longest. Anal margin also with small apical patch of 2–5 fuscous-black spatulate scales in fringes at apex; fringes drab, mixed with white elongate scales and a few fuscous-black scales basad of scale tooth. Ventrally with basal half of first lobe ochraceous-tawny, banded distally by white, russet, white, and ochraceous-tawny respectively; fringes drab or drab mixed with clay or cream color. Second lobe dull russet; venous scales russet or antique brown; fringes drab. Third lobe ochraceous-tawny or russet, with solid white just basad of scale tooth or mixed white and ochraceous-tawny and trail of white bordering anal margin; fringes as on dorsum. ABDOMEN dorsum mixed ochraceous-tawny and clay with varying amounts of chestnut-brown. A white diverging longitudinal subdorsal band present on A1, on posterior half of A2, reduced to patch of white scales on posterior margin of A3–A7. Segments A2–A7 also with small marginal patches of white scales laterally. Males with distinct, mostly pale chestnut-brown, scale tuft laterally on A8, female with banding contiguous from A7. Abdomen venter clay colored, with narrow white mesal band flanked posteriorly by white scale patches. Males with clay colored and white lateral tuft at A8.

**Male genitalia** (Figs 17, 18) (n=5). Uncus mesally articulated with tegumen, length less than half that of tegumen, bilobed, weakly sclerotized except for lobes which bear short setae. Tegumen bilobed; lobes distinctly longer than valvular lobes, with widened base, slightly concave medial margin, and distally truncate. Valvae symmetrical, with terminal membranous valvular lobe accounting for about one-third of valve length. Valvular lobe bearing deciduous scale tuft and short setae, basal connection constricted and inserted just anterad of apex of main part of valve. Sacculus mostly parallel to valve margin, not distinctly wider at base. Juxta a short curved sclerotized fulcrum attached to membranous sheath of phallobase. Phallus slightly curved; length similar to valve length. Phallobase width similar or just exceeding valve width. Aedeagus slightly longer than phallobase, with subapical spinous crest-shaped process. Vesica tubular, without cornuti. Inception of bulbus ejaculatorius at 0.5× length of phallobase. Sternite VIII produced as bilobed structure medially supported by saccus, overall length about one-half that of tegumen, notch between lobes reaching about 0.7× distance to base. Lobes not distinctly widened basally as in *O. eleanerae*.

**Figures 21–26.**
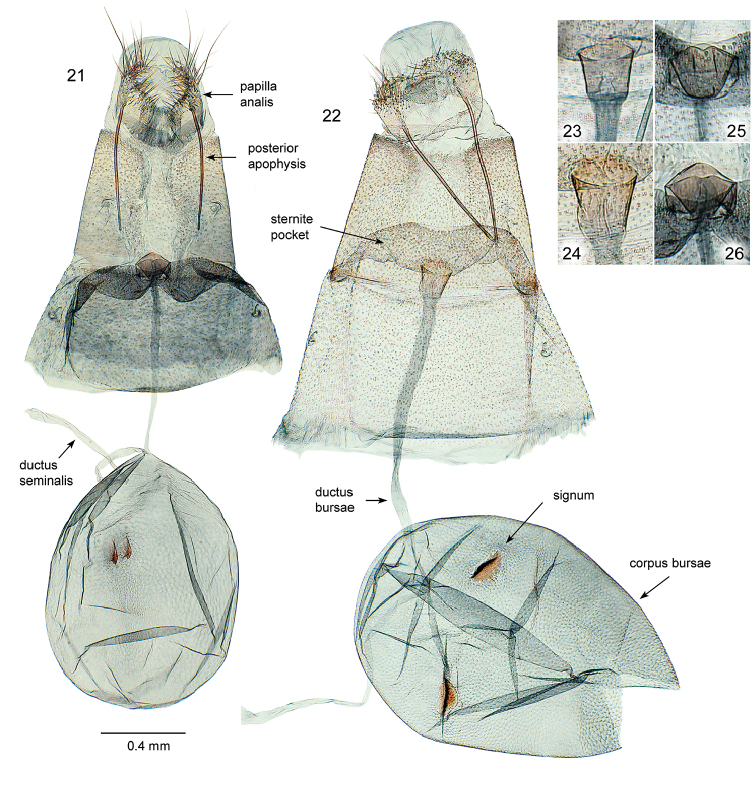
*Oxyptilus* female genitalia: **21** *O. delawaricus*, slide DM 1836, Washington, Chelan County, Wenatchee NF, Swakane Canyon Rd. (FR 7415), 4.2 mi. W of jct w/Hwy 99, 8 Jul 2010, James Adams (DMC) **22** *O. eleanerae* paratype, slide DM 1831, Arizona: Apache County (see material examined) **23–26** enlargements of ostium/antrum - **23** *O. eleanerae* paratype, slide DM 1835, Arizona: Apache County **24** *O. eleanerae* paratype, slide DM 1831, Arizona: Apache County **25** *O. delawaricus*, slide DM 1811, California, El Dorado County, S. Lake Tahoe, Cold Creek, 1 Aug 1977, D.L. Bauer, MGCL Acc. # 2010-29 **26** *O. delawaricus*, slide DM 1836, data cited above for same slide.

**Female genitalia** (Figs 21, 25, 26) (n=5). Apophyses posteriores about 3.5× length of papillae anales, moderately sclerotized. Apophyses anteriores absent. Sternite VII overriding VIII but not extending beyond ostium and forming separate recessed pocket on each side of antrum. Ostium formed from margin of dorsal and ventral antrum plates; dorsal (outer) plate projecting as single rounded medially convex rim; ventral plate weakly bilobed or medially emarginate. Antrum length similar to width, generally appearing funnel-shaped with outer collar where fused with sternite VII. Ductus bursae and corpus bursae similar in length. Width of ductus bursae about 0.2× ostium diameter. Ductus seminalis similar in width to that of ductus bursae; inception point with corpus bursae directly adjacent to that of ductus bursae. Corpus bursae ovoid, with paired, small, serrated blade-like signa, length about 0.6× ostium diameter.

#### Larval hostplants and habits.

Larvae web together leaves and feed within these masses on the developing inflorescences of *Hieracium* (hawkweeds) (Asteraceae). Three species have been recorded as hostplants: *H. abiflorium* Hook., *H. cynoglossoides* Arv.–Touv. (= *H. albertinum* Farr), and *H. scouleri* Hook. (Clarke 1934, Dyar 1904, Landry 1987, McDunnough 1927).

#### Distribution and phenology.

*Oxyptilus delawaricus* occurs in both the western and eastern United States and Canada with a large gap in the distribution representing the Great Plains (Fig. 31). Collection records are concentrated in the Northern and Middle Rocky Mountains and Pacific Northwest, extending south along the Pacific Border into Southern California, and north into the middle of British Columbia. Populations may follow the hostplant range further north and across the northern Interior Plains and Canadian Shield, joined by those known from the Appalachian Highlands and southern Interior Plains. Additional study of material from northern Alberta and Saskatchewan is necessary to determine the northern limits and potential for east-west gene flow within the species.

Distribution records of *O. delawaricus* do not overlap with those of *O. eleanerae* which is restricted to sky islands within the southern part of the Intermountain Basin and Plateau Region. No records of *O. delawaricus* have been found as yet for Colorado, likewise showing a separation of the two species by the Wyoming Basin and Southern Rocky Mountains. Adults have been collected from the middle of May through the first week of September. The earliest confirmed spring records (mid May) are from Northern Mississippi, and are also the southernmost records with dates included on the labels. Late season records (August, September) are from more northern latitudes but no clear flight patterns are evident.

#### Notes.

Zeller (1873) described *O. delawaricus* from a male collected along the Delaware River. Barnes and Lindsey (1921) indicate the holotype should be in the British Museum (Natural History Museum, London). Grinnell (1908) described *Oxyptilus bernardinus* and O. bernardinus form finitimus . Form finitimus was named based on one specimen that differed in the white forewing markings from the rest of the series. Barnes and Lindsey (1921) synonymized *Oxyptilus bernardinus* with *Capperia ningoris* (Walsingham) and *bernardinus* form finitimus with *O. delawaricus*. Barnes and Lindsey (1921) selected and designated a [lecto]type from five of Grinnell’s specimens which is currently in the USNM type collection. They also found an unlabeled specimen with Grinnell’s *bernardinus* series which they recognized as the specimen Grinnell referred to as form finitimus and labelled this specimen “type”. The later specimen, however, has not been found in USNM holdings. Male valvular lobes are easily broken off during dissection and frequently missing on older specimens.

**Figures 27–30.**
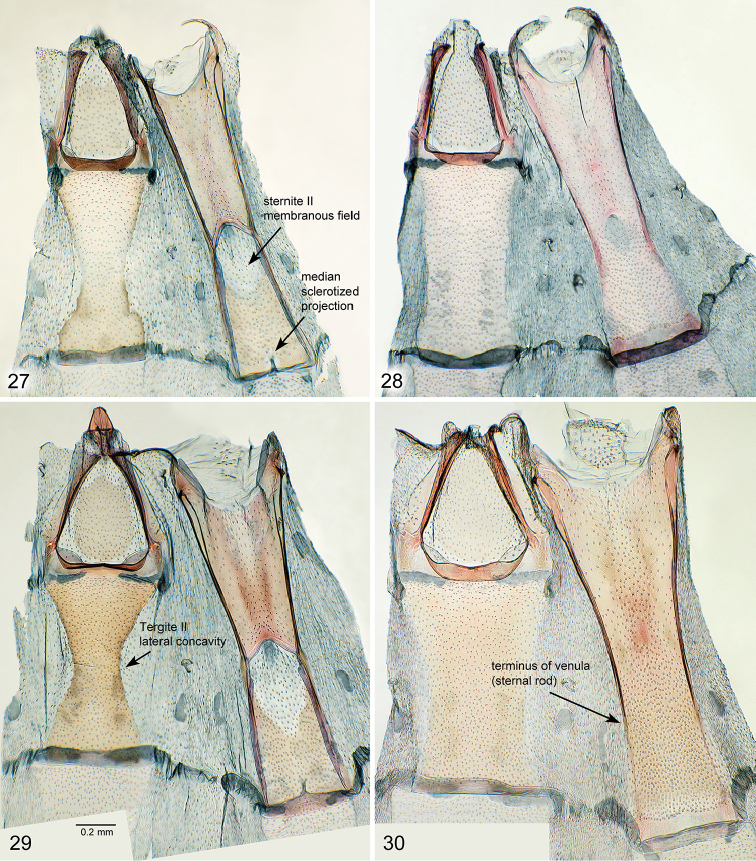
*Oxyptilus* abdominal segments I and II: **27** *O. delawaricus* male, slide DM 1837, California, El Dorado County, S. Lake Tahoe, Cold Creek, 10 Aug 1991, D.L. Bauer, MGCL Acc. # 2010-29 **28** *O. delawaricus* female, slide DM 1836, Washington, Chelan County, Wenatchee NF, Swakane Canyon Rd. (FR 7415), 4.2 mi. W of jct w/Hwy 99, 8 Jul 2010, James Adams; **29** *O. eleanerae* male, slide DM 1829, New Mexico: Sandoval County **30** *O. eleanerae* female, slide DM 1831, Arizona: Apache County.

**Figure 31.**
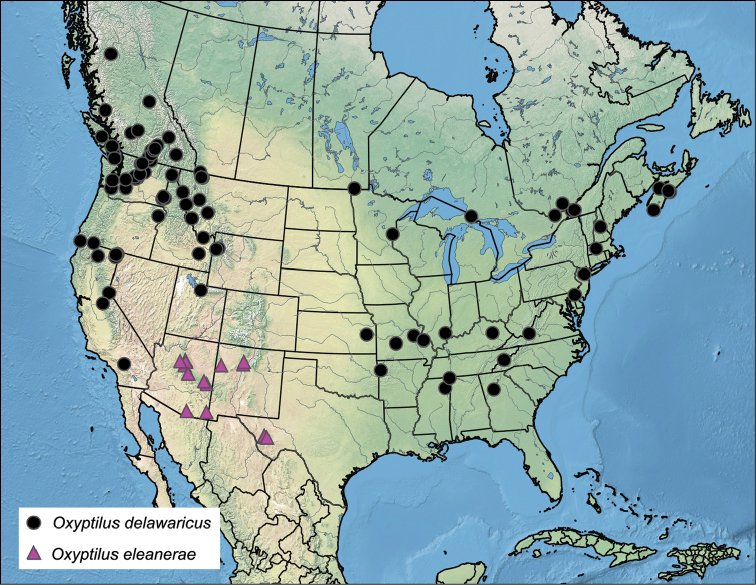
Distribution of *Oxyptilus delawaricus* and *O. eleanerae*. Assembled with simplemappr.net, accessed March 2017.

## Discussion

As a key character, the position of the lateral concavity of tergite II in males, along with other characters of the upper abdominal segments, was of interest in confirming the generic placement of the new species. In males of both *O. eleanerae* and *O. delawaricus* this concavity (Figs 27, 29) is positioned near the middle of the segment, as opposed to more posterior in *Geina* and *Capperia*. In females (Figs 28, 30), the tergite margins are parallel or with a slight central concavity (*O. eleanerae*) similar to males. On sternite II, the venulae (lateral sclerotized ridges or sternal rods), extend to the posterior margin in males but only to about three-fourths to the posterior margin in females of both species. In males, sternite II bears a median sclerotized projection on the posterior margin. This projection is absent in females or extremely reduced. Males of both species have a distinct membranous central field in the posterior half of sternite II which is absent in female *O. eleanerae* and reduced in *O. delawaricus*.

Two of the *O. eleanerae* paratypes (previously sorted in USNM protem material as *Capperia* sp.) bear Barcode of life voucher numbers (see Type material section). The corresponding CO1 sequences were retrieved via the BOLD systems public portal (Ratnasingham and Hebert 2007). The neighbor-joining taxon ID tree obtained from BOLD, based on the Kimura 2 parameter (Kimura 1980) places *O. eleanerae* within *Oxyptilus*; differing from *O. delawaricus* by 2% (about 14 base pairs), and *O. delawaricus* + *O. chrysodactyla* by 1.75%. As in *O. pilosellae*, there is considerable haplotype variation within *O. delawaricus*, approaching 1% between western populations (n=15) and two eastern samples (southeast Manitoba and Illinois). Along with searching for the northern distribution limits of *O. delawaricus* in Canada, additional sequencing of material from the eastern United States and Canada is needed. These sequences will reveal the extent of variation between eastern populations and help determine if there is indeed geographic isolation of haplotypes by the Great Plains, historically the Western Interior Seaway of the Cretaceous Period. No patterns of morphological differences were observed in eastern vs. western *O. delawaricus*. The possibility of host races should be considered along with isolation by geography and glacial maxima, if warranted by additional molecular findings.

The close relationship of *Crombrugghia* and *Oxyptilus* found by Alipanah et al. (2011) based on adult morphology is supported by CO1 barcodes. Within the genus *Oxyptilus*, the close relationship of *O. chrysodactyla* and *O. delawaricus* is likewise supported. Based on CO1, *O. eleanerae* is best placed as a sister species to *O. delawaricus* + *O. chrysodactyla*.

Alipanah et al. (2011) proposed two potential morphology based synapomorphies present in “true *Oxyptilus*” involving the shape of tergite VIII in males (Character 68) and a basal sclerotized process of the valvae (Character 138). These characters were scored as missing at the time (68 in *O. delawaricus* and 138 in *O. chrysodactyla*) because they were either obscured or material was not available. In the present study, Character 138, was examined and found consistent with state 1 (basal sclerotized process triangular) for *O. chrysodactyla* as well as *O. eleanerae*, thus confirming the synapomorphy for Clade N (“true *Oxyptilus*” + *Crombrugghia*) (Alipanah et al. 2011). The character is, however, somewhat difficult to discern depending on the orientation of valvae in preparations. Character 68, shape of eighth tergite, was found to be state 0 (almost semicircular) in both *O. eleanerae* and *O. delawaricus*. This character is thus also confirmed as a synapomorphy for Clade N. Future genomic studies along with morphological studies of immature stages may further clarify relationships within the genus.

In addition to the extent of the northern range of *O. delawaricus*, that of the southern range of *O. eleanerae* is unknown. Examination and identification of material from northwestern Mexico, specifically the southern extent of the Madrean Archipelego (Warshall 1995), will be of interest in determining the full range of this new species. Likewise, the challenge of discovering the life history of *O. eleanerae* is on.

## Supplementary Material

XML Treatment for Oxyptilus eleanerae

XML Treatment for Oxyptilus delawaricus
